# Supplementary figures and images for: Cancer cells with high-metastatic potential promote a glycolytic shift in activated fibroblasts
Source: PLoS One. 2020 Jun 17;15(6):e0234613. doi: 10.1371/journal.pone.0234613 (PMC7299357; doi:10.1371/journal.pone.0234613)

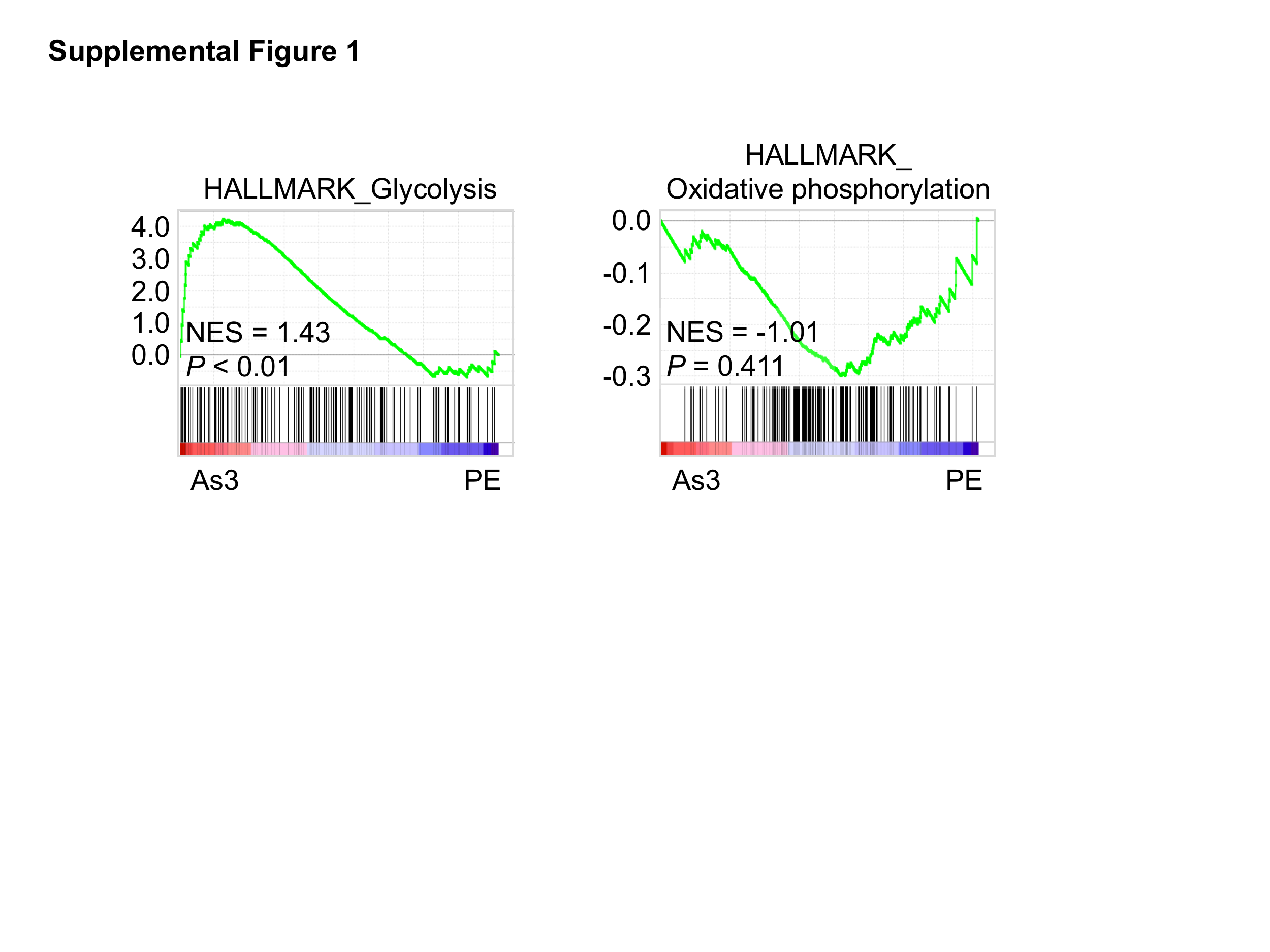

Supplement: S1 Fig — Western blot analysis of glycolysis-related proteins, ENO1, ENO2, LDHA, PDK1, PDK3, and β-actin in the cells cocultured with DGC cells with high metastatic potential or in the mono-cultured iNF60 cells (left). Densitometric analysis of Western blot on ENO2, LDHA and PDK3 normalized to the level of ACTB (right). Error bars represent s.d. *, p < 0.05 from ANOVA followed by Tukey’s HSD post hoc comparisons. (TIFF) [file pone.0234613.s001.tiff]

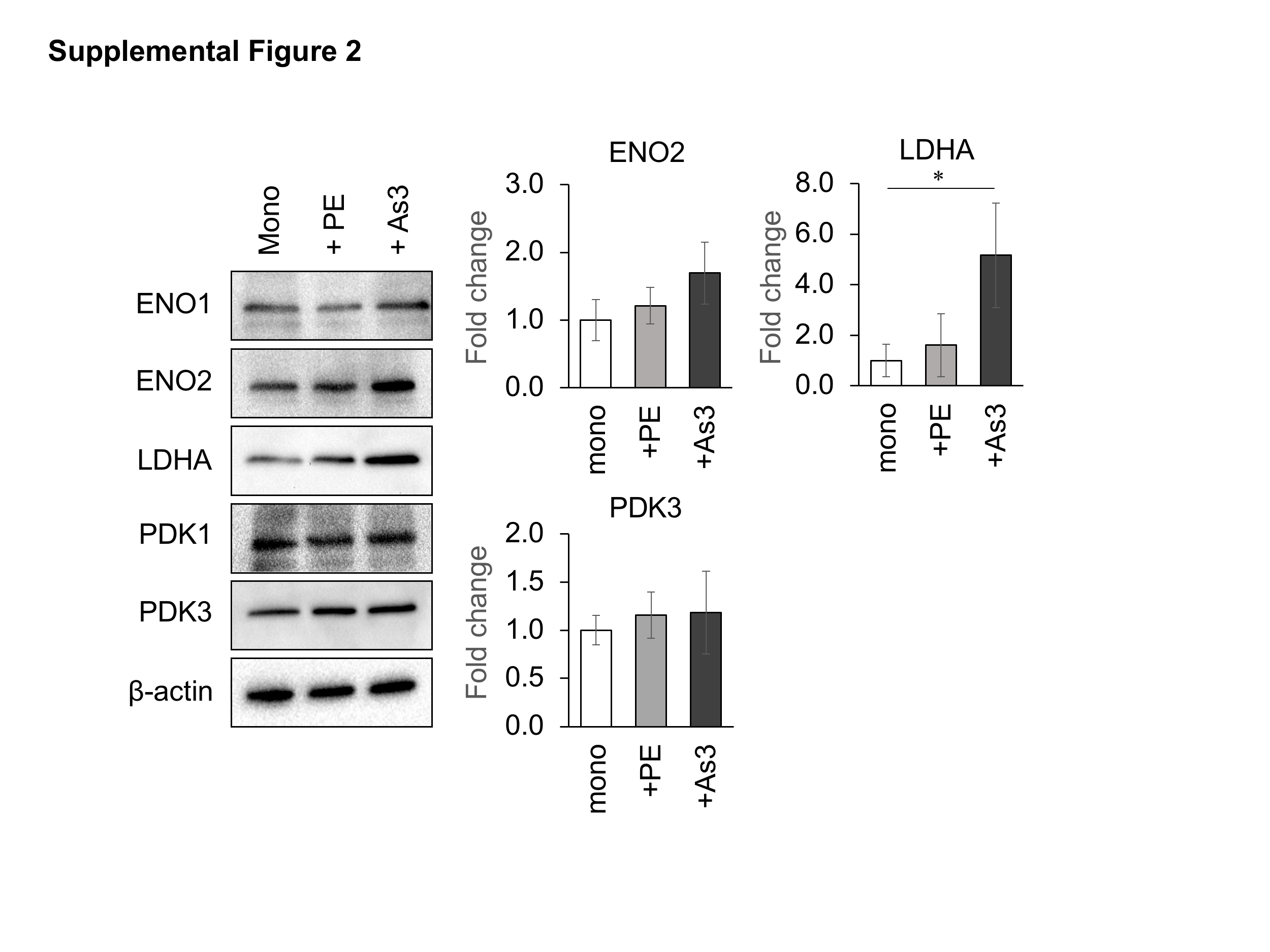

Supplement: S2 Fig — NES: normalized enrichment score. The p-value was calculated by GSEA. (TIFF) [file pone.0234613.s002.tiff]

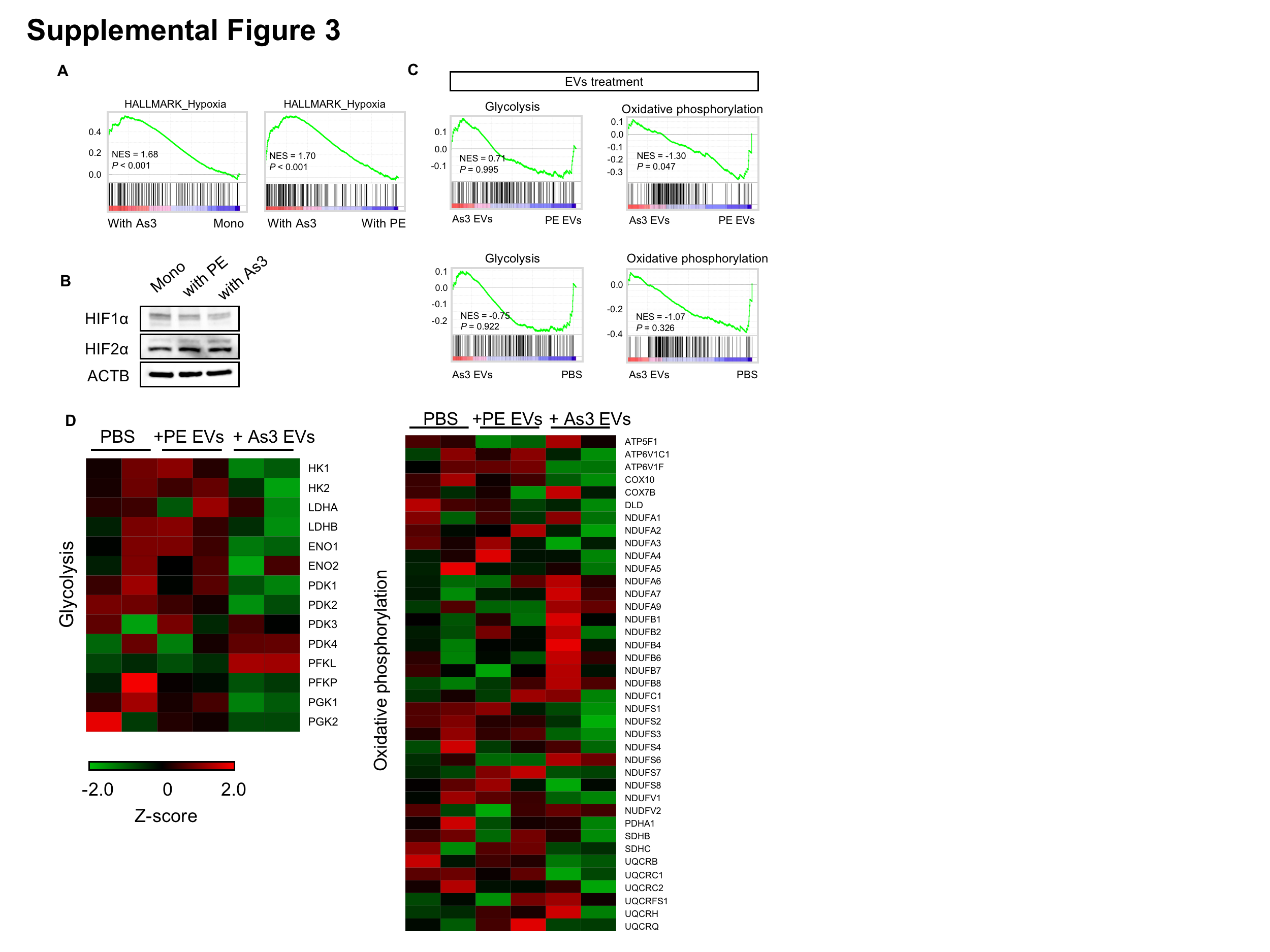

Supplement: S3 Fig — (A) GSEA of the fibroblasts cocultured with 44As3 cells versus mono-cultured fibroblasts (left) and the fibroblasts cocultured with 44As3 cells versus those cocultured with PE (right), highlighting a hypoxia-related phenotype. NES: a normalized enrichment score. The p-value was calculated by GSEA. (B) Western blot analysis of HIF-1α and HIF-2α in fibroblasts cocultured with DGC cells with high metastatic potential or mono-cultured iNF-58 cells. (C) GSEA of the fibroblasts treated with 44As3-derived extracellular vesicles (EVs) (44As3 EVs) versus those treated with HSC-44PE (PE EVs) (upper) and the fibroblasts treated with 44As3-derived extracellular vesicles (44As3 EVs) versus PBS-treated fibroblasts (PBS) (lower), highlighting glucose metabolism and oxidative phosphorylation phenotypes. NES: a normalized enrichment score. The p-value was calculated by GSEA. (D) Heat map showing glycolysis-related and oxidative phosphorylation-related gene expression in each condition. n = 2 technical replicates. (TIFF) [file pone.0234613.s003.tiff]
