# Supplementary material for: Cancer cells with high-metastatic potential promote a glycolytic shift in activated fibroblasts
Source: PLoS One. 2020 Jun 17;15(6):e0234613. doi: 10.1371/journal.pone.0234613 (PMC7299357; doi:10.1371/journal.pone.0234613)
Supplement: S4 Fig — Membranes were often cut to enable blotting for multiple antibodies. The blue dotted squares from raw blots were shown in the main text. (PDF) [file pone.0234613.s004.pdf]

Supplementary Figure 4

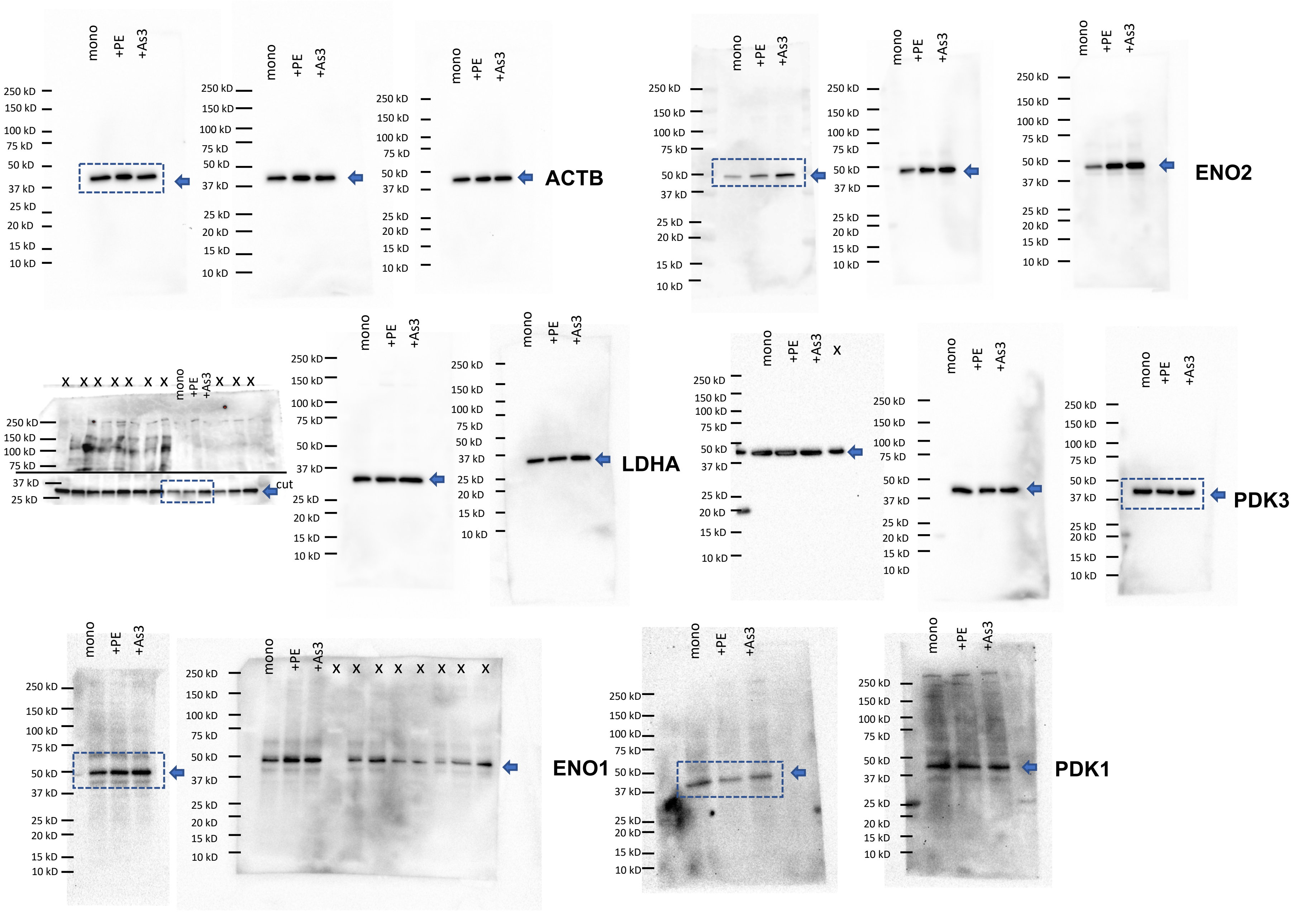

Uncropped full-length pictures of western blotting membranes in Fig.1E.

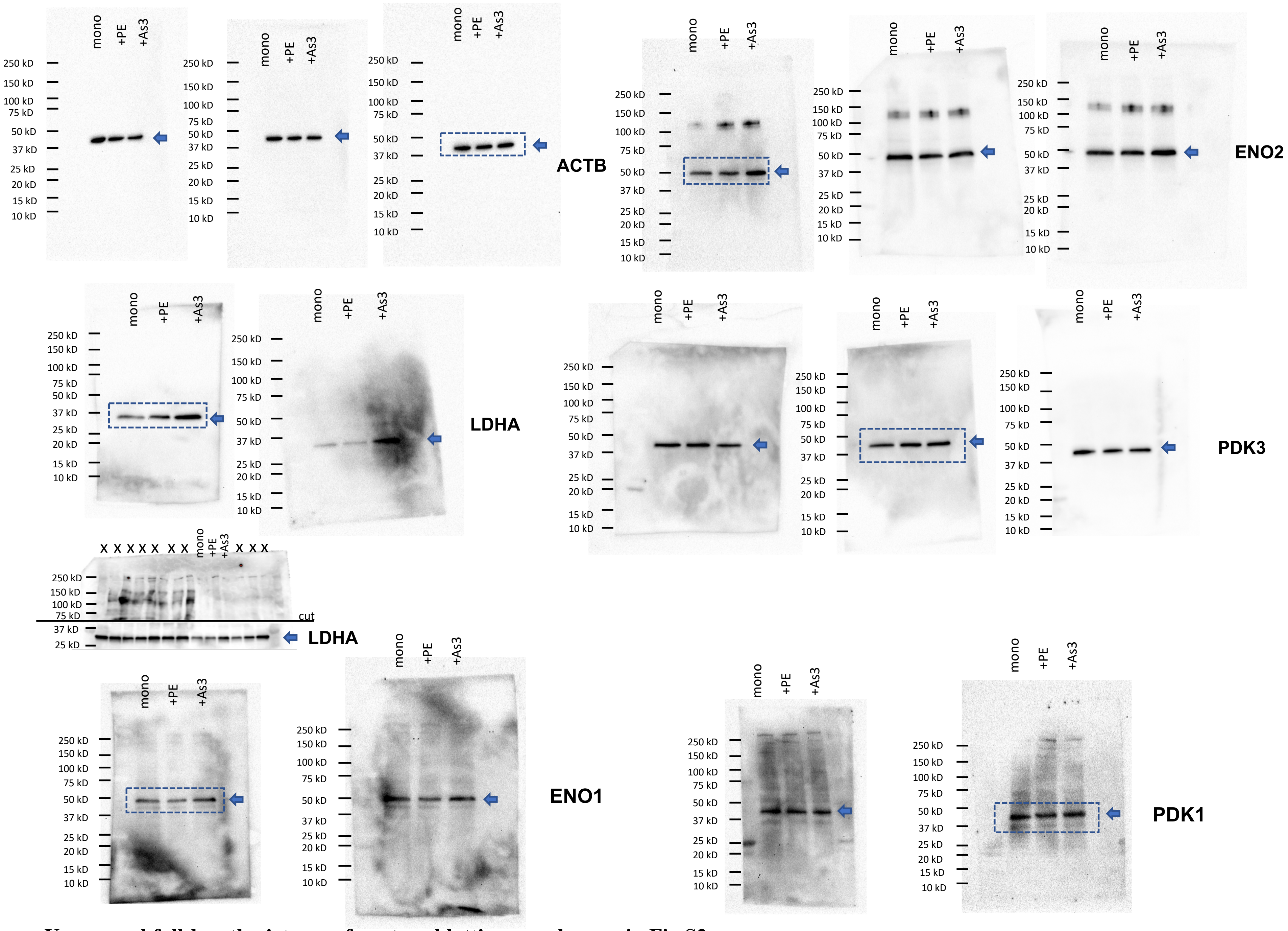

Uncropped full-length pictures of western blotting membranes in Fig.S2.

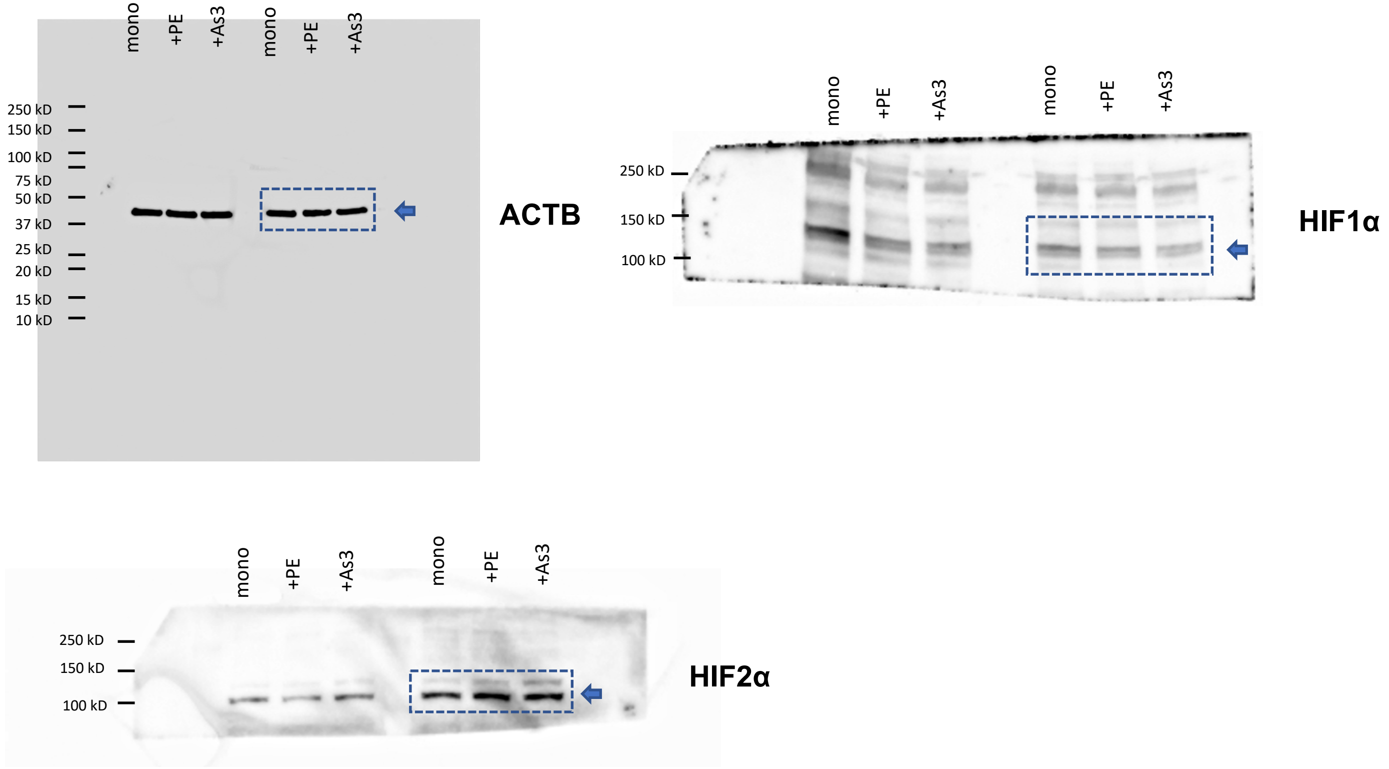

Uncropped full-length pictures of western blotting membranes in Fig.S3.
