# Supplementary material for: Cancer cells with high-metastatic potential promote a glycolytic shift in activated fibroblasts
Source: PLoS One. 2020 Jun 17;15(6):e0234613. doi: 10.1371/journal.pone.0234613 (PMC7299357; doi:10.1371/journal.pone.0234613)
Supplement: Attachment — Submitted filename: Rebuttal.docx [file pone.0234613.s005.docx]

Point-by-point response to each of the reviewer’s comments

We are grateful to all of the reviewers for their critical comments and insightful suggestions that have helped us considerably improve our paper. As indicated in the responses below, we have taken all of these comments and suggestions into account in the revised version of our paper, including the supplementary information. In addition, we included Dr. Kazuyoshi Yanagihara into the author list. Moreover, we added the information about each of the cell lines used in this work in the Materials and methods “Cell cultures”.

**Response to the comments from Reviewer #1**

***Q1. It is unclear how the coculture system employed can be properly interpreted. In Figure 2, lactate in the co-culture system is supplied by both cancer cells and fibroblasts.***

A1. We apologize that we didn’t properly describe it in the text. We applied the transwell culture system for the coculture of the fibroblasts and DGC cells. As the reviewer#1 pointed out, both DGC cells and fibroblasts enabled to supply the lactate with each other during incubation. However, for lactate production assay, we firstly removed the chamber including cancer cells, and washed them with PBS. Then, we replaced fresh media with 1g/L glucose and incubated these fibroblasts cocultured with DGC cells. We measured the lactate level in the cultured media of fibroblasts in 3 hr after the medium change. On these methods, we believe that there is no lactate derived from DGC cells left over. We added these methods in the Materials and Methods section of “Analysis of lactate production, glucose consumption and pH.”

***Q2. Are HSC-44PE cells more glycolytic than 44As3? It is very critical to characterize and present metabolic features (e.g. glycolysis) of cancer cells as well.***

A2. This is an important comment. To address this point, we also run GSEA and found that the "glycolysis" pathway was significantly enriched in 44As3 compared to HSC-44PE (Figure R1). Therefore, it suggests that similar glucose metabolism features of highly metastatic DGC cells were induced in the fibroblasts by coculture. We added these important findings in the Fig S1 and main text (Page 7, line 23 to 26). However, since we would like to focus on the metabolic features on the fibroblasts surrounding cancer cells, we have not performed additional experiments to characterize cancer cells in this manuscript.

Figure R1. GSEA of the 44As3 versus HSC-44PE that were in mono-cultured. NES: normalized enrichment score. The *p*-value was calculated by GSEA.

***Q3. It is also very confusing how the authors performed the Kaplan-Meier survival analysis. It seems that the LDHA and ENO2 expression is not specific to CAFs. If so, how this survival data can support the hypothesis that the authors are proposing?***

A3. We completely agree with reviewer's comment because Kaplan-Meier plotter data are based on gene expression profiles from the bulk tumour tissues. The reviewer #2 also gave us a similar comment. These data do not directly reflect the relationship between patient outcome and expression of LDHA and ENO2 in the fibroblasts within tumour microenvironment. Therefore, we removed Fig.4 from the revised manuscript to avoid misunderstanding for the readers.

***Q4. The previous study published by the same group was cited twice (#15 and 16).***

A4. We do appreciate your comment. We corrected the references.

**Response to the comments from Reviewer #2**

***Q1. Most of the experiments are done with two replicates, the experiments need to be performed with at least three technical replicates.***

A1. This is an important comment. We performed all experiments with at least three technical replicates and we added them in the figures (Fig1 D and E, Fig2 B). Following these changes, we also edited the text in the result section (Page 8, line 27 to 30)

***Q2. In Fig 1B, the GSEA comparisons are made between As3 with PE and As3 with monoculture. The GSEA analysis between PE and monocultures need to be included. What is the status of enrichment in glycolysis and oxidative phosphorylation in PE versus monoculture.***

A2. Thank you very much for your valuable comment. We also performed the GSEA analysis between fibroblasts cocultured with HSC-44PE and those in mono-culture. As shown in Figure R2, GSEA showed that significant enrichment of genes with a role in “glycolysis” functions in fibroblasts cocultured with HSC-44PE cells compared to fibroblasts that were in mono-culture. On the other hand, the fibroblasts cocultured with HSC-44PE showed the tendency to associated with gene enrichment of "oxidative phosphorylation" although there are no significant differences. These results suggest that HSC-44PE also affects glucose metabolism in the fibroblasts. However, "glycolysis" functions were significantly enriched in the fibroblasts cocultured with 44As3 compared with that with HSC44PE, suggesting that different glucose metabolism would be induced in the fibroblasts by high-metastatic and low-metastatic GC cells. We added these data in Fig.1B and edited the text in the result part (Page 7, line 17 to 19)

Figure R2. GSEA of the fibroblasts cocultured with 44As3 versus those cocultured with HSC-44PE and the fibroblasts cocultured with 44As3 versus mono-cultured fibroblasts and the fibroblasts cocultured with HSC-44PE versus mono-cultured.

***Q3. In Fig1E, the densitometric analysis of blots needs to be added to highlight the differences. The authors should repeat the co-culture experiment with other fibroblast cells (iNF-58).***

A3. We do appreciate your comment. We performed western blot by using iNF-58 and iNF-60. Then, ENO2, LDHA, and PDK3 expressions were evaluated by densitometric analysis. As shown in Figure R3, ENO2 and LDHA expressions were significantly increased in the iNF-58 cocultured with 44As3 cells. On the other hands, in iNF-60 with 44As3, only LDHA expression was increased. This observation might be due to the derived from different patient of iNF-58. These data were added in the revised Fig.1E and Fig.S2, and main text (Page 7, line 29 to 30).

Figure R3. Western blot and densitometric analysis of glycolysis-related proteins in (A) iNF-58 and (B) iNF-60 in mono-culture or coculture with DGC cells. Error bars represent s.d. *, *p* < 0.05, **, *p* < 0.01 from ANOVA followed by Tukey’s HSD post hoc comparisons.

***Q4. In Fig2C, the cell growths should be compared with the monocultures of fibroblasts and cancer cells. Also, the statistics is missing in the figure.***

A4. Thank you very much for your valuable comment. We also checked the proliferation rate in both DGC cells and fibroblasts that were in mono-culture and compared with those in co-culture. As shown in Figure R4, the coculture did not promote cell growth in the fibroblasts (Figure R4A). The proliferation rate of mono-cultured 44As3 was higher than HSC-44PE (Figure R4B). However, there is no significant difference between HSC-44PE grown with fibroblasts and 44As3 grown with fibroblasts. These data suggest that there is no relevance between glycolysis induction in the fibroblasts by DGC cells and cell growth. We added these data in the revised Fig.2C and edited the text in the result section (Page 8, line 32 to 33, and Page 9 line 1 to line 8).

Figure R4. The cell proliferation rate of iNF-58 cells (left) and DGC cell lines (right) in the mono-culture and coculture. n = 3 technical replicates. Error bars represent s.d. *, *p* < 0.05 from ANOVA followed by Tukey’s HSD post hoc comparisons.

***Q5. The authors have correlated levels of genes Ldha and Eno2 with survival of GC patients. However, it will be pertinent to show the expression of Ldha and Eno1 in fibroblast in stromal cells of cancer patient's specimens. Also, it will be interesting to note if there is any survival difference between the patients classified on the basis of glycolytic gene expression in stromal cells.***

A5. According to the reviewer #1’s comment, Kaplan-Meier plotter data are based on gene expression profiles from the bulk tumour tissues. These data do not reflect the relationship between patient outcome and expression of LDHA and ENO2 in the fibroblasts. Since GC tissue samples were limited, it was difficult to use the samples for an immunohistochemistry analysis. Therefore, we removed Fig.4 from the revised manuscript.
